# The influence of renewable energy usage on consumption-based carbon emissions in MINT economies^☆^

**DOI:** 10.1016/j.heliyon.2022.e08941

**Published:** 2022-02-16

**Authors:** Tomiwa Sunday Adebayo, Abraham Ayobamiji Awosusi, Husam Rjoub, Ephraim Bonah Agyekum, Dervis Kirikkaleli

**Affiliations:** a Department of Business Administration, Faculty of Economics and Administrative Science, Cyprus International University, 99040 Nicosia, Turkey; b Department of Finance & Accounting, Akfa University, Tashkent, Uzbekistan; c Faculty of Economics and Administrative Science, Department of Economics, Near East University, Northern Cyprus, TR-10 Mersin, Turkey; d Department of Accounting and Finance, Faculty of Economics and Administrative Sciences, Cyprus International University, Mersin 10, 99040 Haspolat, Turkey; e Department of Nuclear and Renewable Energy, Ural Federal University Named after the First President of Russia Boris, 19 Mira Street, Ekaterinburg, 620002 Yeltsin, Russia; f Faculty of Economics and Administrative Sciences, Department of Banking and Finance, European University of Lefke, Northern Cyprus TR-10, Mersin, Turkey

**Keywords:** Consumption-based carbon emissions, Globalization, Renewable energy, Economic growth, MINT Economies

## Abstract

An accurate carbon emissions measurement is critical for developing an appropriate climate strategy to address ecological issues. A meaningful climate policy reaction can be offered based on trade adjusted statistics of carbon emissions. This research utilizes second-generation panel co-integration techniques to investigate the influence of globalization and renewable energy utilization on consumption-based carbon emissions (CCO_2_) as well as the role of nonrenewable energy use and economic growth in the MINT-(Mexico, Indonesia, Nigeria and Turkey) countries from 1990 to 2018. The outcomes of the cross-sectional dependency and heterogeneity tests revealed slope heterogeneity and cross-sectional units across nations. Furthermore, the outcomes of the cointegration test provided evidence of a long-run association between consumption-based CO_2_ emissions (CCCO_2_) and the regressors. Moreover, the outcomes of both common correlated effect mean group (*CCEMG*) and augmented mean group (AMG) unveiled that economic growth and nonrenewable energy utilization contribute to the degradation of the environment, while globalization and renewable energy utilization help to curb the degradation of the environment. Furthermore, the outcomes of the causality test showed that all the regressors can predict CCO_2_ emissions in the MINT nations. Thus, policy channeled towards globalization, economic growth, and renewable energy utilization will have a significant effect on CCO_2_ emissions. Based on the study outcomes, significant policy recommendations are made for policymakers in the MINT nations.

## 1 Introduction

One of the most pressing issues confronting modern society is ecological deterioration. Because of its impact on billions of human lives, the topic of environmental damage has garnered considerable attention from both researchers and policymakers [1]. Greenhouse gas emissions (GHGs) are universally recognized as a contributor to global warming [2, 3]. Carbon dioxide (CO_2_) accounts for around 75% of worldwide GHGs emissions. Global climate change and severe weather conditions including, floods, droughts, heatwaves, and heavy rains have become common in the recent decade due to rising CO_2_ levels [4, 5, 6]. Extreme events have a significant influence on people's ecosystems and lives [7, 8]. Various agreements have been reached to minimize ecological impacts including global warming, including the Kyoto Protocol in 1997 the Paris Climate Agreement (PCA) in 2015 and the recent COP 26^1^ in Glasgow in 2021. These agreements are focused on keeping global warming below 1.5 degrees Celsius. Governments around the world are promoting energy-efficient systems to reach this goal. Notwithstanding these accords, global temperatures are rising, and CO_2_ emissions increased at a record high of 2.7 percent in 2018, prompting environmentalists, policymakers, and scholars to identify the critical factors and sources influencing the emissions of CO_2_.

**Figure 1:**
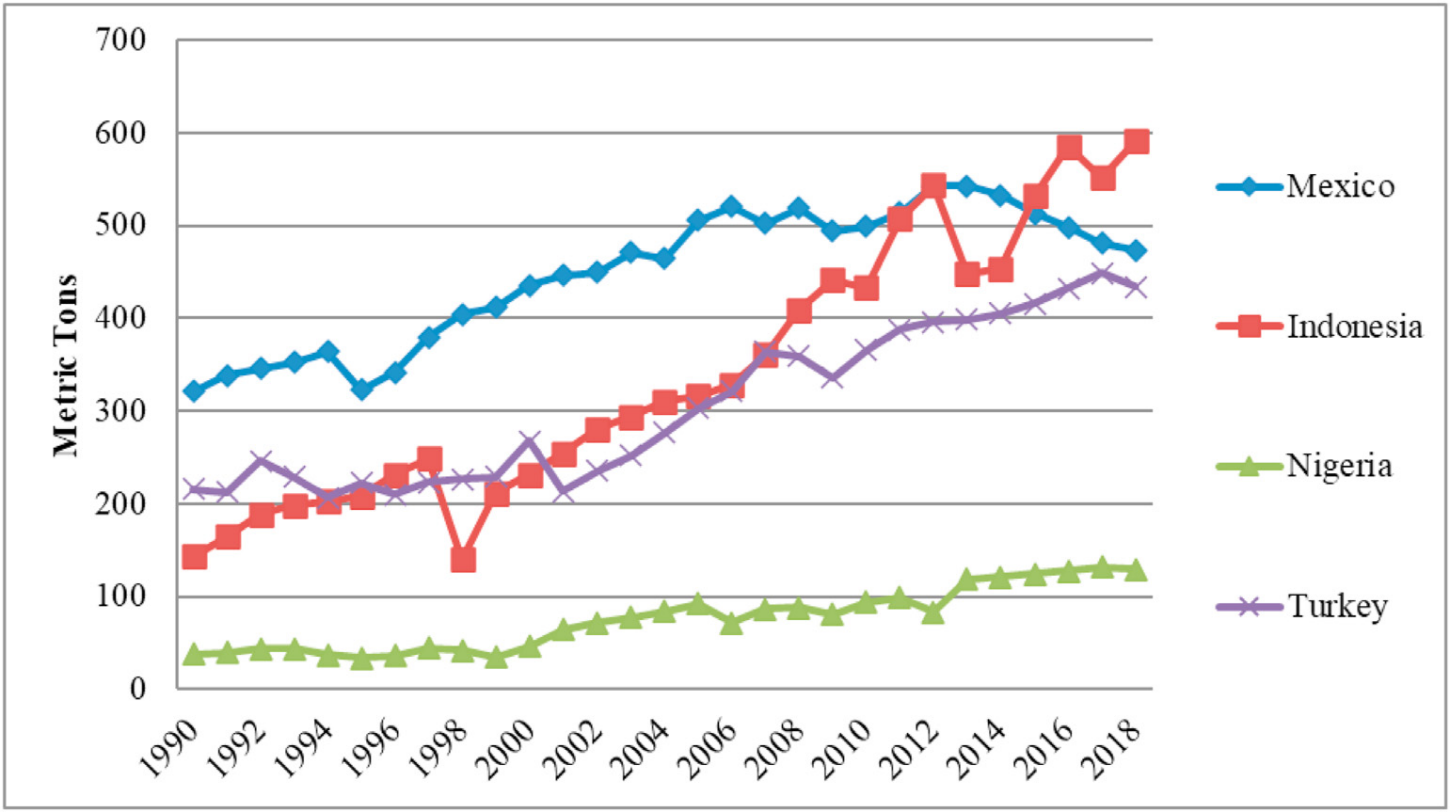
Trends of Consumption-based Carbon emission.

The trend of CO_2_ emissions is disturbing, especially in developing market economies like Mexico, Indonesia, Nigeria, and Turkey, which are collectively known as the MINT nations. Emerging market economies, according to a study issued by the IPCC, are said to emphasize economic expansion over ecological well-being, since these countries have increased their economies whilst contributing over 76.6 percent of world greenhouse gas (GHG) emissions, notably CO_2_ [9]. In general, industrialized nations are more likely to produce the majority of global GHGs, although emerging country emissions have also grown in recent years [10]. The world has increased its focus on the BRICS (Brazil, Russia, India, China, and South Africa) economies, a powerful emerging group in the developing world. Other emerging markets, including Mexico, Indonesia, Nigeria, and Turkey (MINT), were also identified by O'Neill in 2013. As stated by [11], MINT nations account for roughly 1%–2% of the global economy and have a strong likelihood of becoming the globe's largest economies in the following decades, both economically and technologically. As per Gold Sachs, the MINT nations will have a steady growth trajectory [12]. Table 1 illustrates the ratio and difference between consumption-based (CCO_2_) and territory-based carbon (TCO_2_) emissions for the MINT nations. Table 1 shows that Indonesia and Nigeria are exporters of emissions, while both Mexico and Turkey are importers of carbon emissions. Figure 1 also shows the trend in CO_2_ emissions caused by consumption in the MINT countries.

**Table 1:** Consumption and Terrestrial based emissions in MtCO_2_.

Country	$\frac{\mathrm{CC}O_{2}}{\mathrm{TC}O_{2}}$	$\mathrm{CC}O_{2}-\mathrm{TC}O_{2}$	Outcome
Mexico	1.05803	24.5618	Import carbon emissions
Indonesia	0.96840	-11.0366	Export carbon emissions
Nigeria	0.90339	-8.08227	Export carbon emissions
Turkey	1.13367	35.9265	Import carbon emissions

The increased use of energy has a significant impact on the quality of the environmental and emissions of CO_2_ [13,14]. Renewable energy (including solar, tidal, geothermal, wind power, biomass, and hydro) produces lower emissions than fossil fuels, which are regarded to be the primary cause of global warming and CO_2_ emissions [15, 16]. As a result, one of the most significant methods of reducing emissions of CO_2_ is to employ renewable sources of energy [17]. Following the renowned Kyoto Protocol in 2005 and the United Nations Conference on Climate Change (COP-21), the utilization of renewable energy sources has become a propelling tactic for advanced nations seeking to meet their target of reducing GHGs emissions. A number of studies have included renewable energy consumption as a significant variable in CO_2_ emissions regressions due to its relevance in reducing CO_2_.

Globalization is a worldwide phenomenon that significantly affects people's political, economic, and social lives. It lowers/eliminates cross-border obstacles, facilitates contemporary technology transfers, and increases capital inflows investment [18]. Although globalization has a positive impact on the economy, it also has a negative impact on the environment [2, 19]. The impact of globalization on CO2 emissions is theoretically unclear. As a consequence, there is ongoing debate over the globalization-emissions interrelationship. For instance, some studies have found a negative relationship between the two variables [2, 19, 20, 21], while others have found a positive relationship [22, 23, 24]. This leads to the questions, do globalization and renewable energy consumption mitigate emissions, and do growth and nonrenewable energy use contribute to environmental degradation? Therefore, considering the potential environmental consequences of globalization and the utilization of energy (renewable and nonrenewable energy), this study investigates the dynamic effects of globalization and the utilization of energy on CO_2_ emissions in the MINT nations as well as the influence of economic growth.

The contributions of this research are as follows: First, even though several prior studies have used time-series data and panel analysis to examine the impact of globalization on CO_2_ emissions and ecological footprints, panel research on the impact of globalization on consumption-based CO_2_ emissions has yet to be conducted. Secondly, as a measure of environmental deterioration, we used CCO_2_ emissions, which is international trade-adjusted pollution. The literature, on the other hand, has mostly concentrated on CO_2_ emissions based on region. According to recent research, it is preferable to examine consumption-based carbon emissions^2^ rather than territorial-based CO_2_ [25,26]. Second, rather than using the usual method of assessing the environmental impact of aggregate energy usage, this research separates the effects of different energy supplies (renewable and non-renewable) on CCO_2_ emissions in the context of the MINT countries. Disaggregating energy usage is essential since nonrenewable and renewable energy consumption influence environmental quality differently [23, 27].

Third, previous research has mostly focused on the individual effects of globalization and renewable and nonrenewable energy usage on CO_2_ emissions [7, 28, 29]; however, there has been minimal research into the potential combined effects of these factors. As a result, this research examines the interaction effects of globalization, renewable and non-renewable energy consumption on the MINT nation's CCO_2_ emissions to reveal further policy implications. Lastly, we utilized both Augmented Mean Group (AMG) and Common Correlated Effect Mean Group (CCEMG) approaches suggested by [30] and [31], respectively to identify the influence of globalization and renewable energy on Consumption-based CO_2_. These approaches are crucial for a variety of reasons. Firstly, they function in the presence of heterogeneity, endogeneity, cross-sectional dependence heterogeneity, and non-stationarity. Furthermore, these approaches deal with correlation, particularly amongst cross-sections.

The next section presents the theoretical framework and literature review, which is followed by the data and methodology in Section 3. The findings and discussion are presented in Section 4 and Section 5 concludes the study.

## 2 Theoretical framework and literature review

### 2.1 Theoretical framework

Over time, the investigation of the energy–economic–environmental paradox has intensified, dividing the literature into three streams. The first line of research assesses the linkage between greenhouse gas (GHGs) emissions and economic growth (GDP) via the prism of the hypothesis developed by [32] known as the Environmental Kuznets Curve (EKC) hypothesis. According to this hypothesis, GDP is initially related to environmental deterioration, but as the economy experiences continued growth in the subsequent phase, the trade-off gradually lessens, therefore improving the quality of the environment [33]. In terms of the scale and composition effects, the early growth in the economy seems to have an adverse role on the environment, demonstrating that as the economy expands, its production method expands to employ more pollution-intensive equipment, culminating in environmental degradation [34]. The stage at which economic growth complements environmental quality, called the technique effect, is where technological innovation permits economic advancement without negatively impacting the environment [35]. Consequently, the EKC theory has been often criticized for its narrow focus on economic growth as the only cause of environmental deterioration.

The second line of investigation on the energy–economy–environment connection examines how energy use impacts the quality of the environment, and more significantly, the association between energy consumption and environmental quality. Energy is seen as a major element in the process of manufacturing, and a rise in energy consumption is predicted to boost economic production [36]. Similarly, increasing energy consumption will have a negative impact on environmental quality since the combustion of energy resources, principally fossil fuels (coal, gas, and oil), leads to the emission of GHGs; ultimately, increasing energy consumption may be considered to be detrimental to the environment [2]. Conversely, renewable energy consumption is seen as a viable substitute for fossil fuels, which will help to alleviate energy-associated environmental issues. Furthermore, it is expected that by incorporating renewable energy into the energy basket, progressive reductions in fossil fuel dependency will be accomplished, allowing for environmentally sustainable growth.

Globalization is acknowledged as a key element influencing economic growth and energy consumption. Globalization is seen as a strategy for attaining economic growth since it enables a local economy to engage in foreign trade, attracting investors to supply Foreign Direct Investment (FDI) to boost their economic activities, and also provides integration with the rest of the globe via a variety of different outlets. The impact of globalization on the environment remains unclear. It has been argued that globalization has contributed to environmental deterioration by encouraging the expansion of polluting industries in underdeveloped countries [23]. Conversely, globalization-induced foreign trade may be used to specialize in the development of cleaner production processes, such that globalization can be considered to benefit both the economy and the environment. Moreover, depending on whether the FDI is dirty or clean, the consequences of the flow of FDI are unclear. Based on the above theoretical knowledge, we propose the following economic function as indicated in Eq. (1):

(1) $$\mathrm{CC}O_{2}=f\left(\mathrm{GDP}\text{,}\;\mathrm{REN}\text{,}\;\mathrm{NREN}\text{,}\;\mathrm{GLO}\right)$$

Where: CCO_2_, GDP, NREN, REN, and GLO stand for consumption-based carbon emissions, economic growth, nonrenewable energy, renewable energy, and globalization, respectively.

### 2.2 Literature review

Extensive research has been conducted to investigate the drivers of environmental degradation for specific nations or regions. For instance [37], evaluated the interconnectedness of CCO_2_-GDP-REN in India, utilizing quarterly data covering the period between 1990 and 2015. The authors found that the interaction between CCO_2_ and GDP is positive but insignificant, whereas a negative connection was found between REN and CCO_2_ using the DOLS and FMOLS approaches. Conversely, the study of [19] uncovered a positive and significant interconnection between CCO_2_ emissions and GDP over the period 1990–2018 in Mexico employing the dual adjustment approach. Similarly, the study of [38] also confirmed a direct interconnection between CCO_2_ and GDP in 20 Asian nations over the period from 1990 to 2013. For the MINT economies, Adebayo & Rjoub [39] used AMG and CS-ARDL to analyze a dataset ranging from 1990 to 2017 and affirmed a positive interconnectedness between CCO_2_ and GDP. However [26], used a quarterly dataset covering the period between 1990Q1 and 2017Q4 and discovered a positive relation between CCO_2_ and GDP in China, indicating that an upsurge in GDP will result in a rise in CCO_2_ in China using the DOLS, CRR and FMOLS techniques. Hasanov et al. [40] studied the interconnectedness between CCO_2_ and GDP utilizing the CCEMG, PMG, FMOLS and DOLS approaches for oil-exporting nations from 1995–2013 and confirmed that the relationship between CCO_2_ and GDP is positive.

Using the CCEMG and AMG approach, the research of [8] on the G7 nations covering the period between 1990 and 2019 confirmed a positive interconnectedness between CCO_2_ and GDP. Khan et al. [41] discovered a positive interconnection between CCO_2_ and GDP between 1990 and 2018 in nine oil-exporting nations. Knight and Schor [42] examined the connection between CCO_2_ and GDP in twenty-nine high-income nations over the period between 1991 and 2008. The authors found that GDP positively influences CCO_2_. Using the NARDL, the study conducted on Chile by Adebayo et al. [43] over the period from 1990 to 2018 found that both negative and positive variations in GDP contribute to CCO_2_ in Chile. For Japan, the investigation of [44] using quarterly data ranging from 1990Q1-2015Q4 established the presence of a positive interconnectedness between CO_2_ and GDP. These findings were corroborated by the research of [1] in Japan over the timespan from 1970 to 2015, whereas the study performed by [5] on Japan affirmed the validity of the EKC over the period of 1965–2018 using the DOLS and FMOLS approaches. This result was corroborated by the research of Fatima et al. [45] in eight nations over the timespan from 1980 to 2014. Many researchers have discovered that GDP has an adverse impact on CO_2_ in Nigeria [13, 46, 47].

Based on the preceding discussion, non-renewable energy consumption (NREN) helps in economic expansion; however, the consumption of NREN contributes to the emission of pollutants thereby leading to the deterioration of the environment. Over the years, various studies have evaluated the impact of non-renewable energy on the quality of the environment for specific nations or regions. For example [35], examined the connection between NREN and CO_2_ in Latin American economies over the period of 1980–2017 and confirmed that a continuous increase in NREN adds to the increase in carbon emission, leading to the deterioration of the environment. Employing the PMG approach, the research conducted on the G7-economies by [48] from 1990 to 2019 established that the effect of NREN contributes to CO_2_. Mahalik et al. [49] analyzed the connection between CO_2_ and NREN in the BRICS economies over the period between 1990 and 2015 and the authors disclosed that the impact of NREN on CO_2_ is positive. For 32 nations [50], utilized the GMM approach to analyze a dataset ranging from 1996 to 2014 and confirmed a positive interconnectedness between CO_2_ and NREN.

The study of [51] examined the period between 1980 and 2014 and found a positive relation between CO_2_ and NREN in eight nations. Xie and Liu [52] discovered a positive relation between CO_2_ and NREN in China utilizing a dataset covering the period between 1965 and 2016; their findings indicated that an upsurge in the usage of NREN results in an increase in CO_2_ in China. However, the study of [53] confirmed a similar outcome in China using the ARDL approach over the period between 1980 and 2014. Using the GMM, the study of [54] on 10 African nations confirmed a positive interconnectedness between CO_2_ and NREN. The research of [55] also revealed a positive interconnectedness between CO_2_ and NREN over the period between 1970 and 2012 in Pakistan.

To mitigate environmental degradation and also achieve sustainable growth, scholars have argued that renewable energy (REN) could be a good determinant in achieving this goal. Khan [56] utilized the DOLS, CRR, and FMOLS approaches to analyze a dataset ranging from 1990Q1-2017Q4 and the outcomes confirmed a negative interconnectedness between CCO_2_ and REN. Similarly, for the G7 economies [57], and Ding et al. [58] found a negative CCO_2_-REN interconnectedness; however, the research of [48] studied the interconnectedness between CCO_2_ and REN utilizing the PMG approach for the period of 1990–2019 and confirmed that a negative relation exists CO_2_ and GDP in the G7 economies. Furthermore, the research of [33] affirmed that a negative but insignificant connection exists between REN and CO_2_ in South Africa utilizing a dataset from 1980 to 2017. Using the FMOLS, the study of [59] for five EU- nations covering the period between 1990 and 2015 confirmed a negative interconnectedness between CO_2_ and REN. The studies of [37] and [2] on Japan and Argentina, respectively, confirmed a negative interconnectedness between CO_2_ and REN.

Lastly, globalization (GLO) is another determinant of environmental degradation. The study of [49] on the emissions-globalization nexus using data from 1990 to 2015 affirmed a positive interconnectedness between CO_2_ and GLO. Conversely [60], examined the connection between CO_2_ and GLO in 45 Asian nations over the period between 1990 and 2017 and uncovered that GLO negatively influences CO_2_. Also, the research of [61] probed into the interconnectedness between CO_2_ and GLO utilizing the FMOLS approach for the period between 1970 and 2016. The outcomes from this study confirmed a negative relationship between CO_2_ and GLO in the case of the OECD economies. Applying the dual adjustment method, the study of [19] on Mexico found a negative interconnectedness between CCO_2_ and GLO (see Table 2).

**Table 2:** Overview of Literature review.

Scholars	Country of study	Period	Methodology	Outcome(s)
**Environmental degradation and GDP**				
Kirikkaleli & Adebayo [37]	India	1990Q1-2015Q4	DOLS and FMOLS	GDP ≠ CCO_2_ (+)
He et al. [19]	Mexico	1990–2018	Dual adjustment approach	GDP → CCO_2_ (+)
Udemba et al. [62]	Chile	1990–2018	NARDL	GDP^+^ → CCO_2_ (+) GDP^−^ → CCO_2_ (+)
Liddle [38]	20 Asian Nations	1990–2013	CCEMG	GDP → CCO_2_ (+)
Adebayo & Rjoub [39]	MINT	1990–2017	AMG and CS-ARDL	GDP → CCO_2_ (+)
Khan et al. [63]	China	1990Q1-2017Q4	DOLS, CRR and FMOLS	GDP → CCO_2_ (+)
Hasanov et al. [40]	Oil exporting Nations	1995–2013	CCEMG, PMG, FMOLS and DOLS	GDP → CCO_2_ (+)
Ding et al. [58]	G7 Nations	1990–2017	CCEMG, AMG and DH causality approach	GDP → CCO_2_ (+)
Khan et al. [41]	9 Oil exporting Nations	1990–2018	CCEMG, AMG and CS-ARDL	GDP → CCO_2_ (+)
Knight & Schor [42]	29 high-income countries	1991–2008	POLS	GDP → CCO_2_ (+)
Adebayo & Kirikkaleli [1]	Japan	1990Q1-2015Q4	Wavelets tools	GDP → CO_2_ (+)
Awosusi et al. [5]	Japan	1965–2019	DOLS and FMOLS	EKC is valid
Awosusi et al. [33]	Brazil	1965–2019	DOLS, ARDL and FMOLS	GDP → CO_2_ (+)
Ayobamiji & Kalmaz [46]	Nigeria	1971–2015	FMOLS, ARDL and DOLS	GDP → CO_2_ (+)
Awosusi et al. [34]	South Korea	1965–2019	FMOLS, ARDL and DOLS	GDP → CO_2_ (+)
Fatima et al. [51]	8 Nations	1980–2014	GMM	EKC is valid
**Environmental degradation and Non-renewable energy usage**				
Ramzan et al. [35]	Latin America countries	1980–2017	DOLS and FMOLS	NREN → CO_2_ (+)
Ibrahim et al. [48]	G7-Countries	1990–2019	PMG	NREN → CO_2_ (+)
Mahalik et al. [49]	BRICS	1990–2015	GMM	NREN → CO_2_ (+)
Liu et al. [65]	China	1965–2016	ARDL	NREN → CO_2_ (+)
Adedoyin et al. [50]	32 countries	1996–2014	GMM	NREN → CO_2_ (+)
Chen et al. [53]	China	1980–2014	ARDL and VECM	NREN → CO_2_ (+)
Dogan & Inglesi-Lotz [54]	10 African Nations	1980–2011	DOLS	NREN → CO_2_ (+)
Zhang et al. [55]	Pakistan	1970–2012	FMOLS, CCR, ARDL and DOLS	NREN → CO_2_ (+)
**Environmental degradation and Renewable energy usage**				
Kirikkaleli & Adebayo [37]	India	1990Q1-2015Q4	DOLS and FMOLS	REN → CCO_2_ (-)
Udemba et al. [62]	Chile	1990–2018	NARDL	REN^+^ → CCO_2_ (-) REN^−^≠ CCO_2_ (+)
Umar et al. [57]	G7 nations	1990–2017	CCEMG, AMG and DH causality approach	REN → CCO_2_ (-)
Ding et al. [58]	G7 nations	1990–2018.	AMG and DH causality approach	REN → CCO_2_ (-)
Ibrahim & Ajide [48]	G7-Countries	1990–2019	PMG	REN → CO_2_ (-)
Yuping et al. [2]	Argentina	1970–2018	ARDL	REN → CO_2_ (-)
**Environmental degradation and Globalization**				
He et al. [19]	Mexico	1990–2018	Dual adjustment approach	GLO → CCO_2_ (-)
Akinsola et al. [66]	Argentina	1980–2017	DOLS, ARDL and FMOLS	GLO → CO_2_ (-)
Haseeb et al. [67]	South Asian Nation	1985–2018	FMOLS	GLO → CO_2_ (+)
Yang & Zhoa [61]	OECD Nations	1971–2016	FMOLS and DOLS	GLO → CO_2_ (-)
Zafar et al. [60]	45 Asian Nations	1990–2017	FMOLS and DH causality approach	GLO → CO_2_ (-)

Note: CO_2_: Carbon emission; PR: Political risk; REN: Renewable energy consumption, GDP: Economic Growth; NREN: Non- Renewable Energy Consumption; → (+): Positive relationship; → (-): Negative relationship; →: One-way causality; ↔: Two-way causality; T-Y: Toda-Yamamoto causality; D-H: Dumitrescu and Hurlin causality; VECM: Vector Error Correction Model; ARDL: Autoregressive Distributive Lag model; NARDL: Non-linear Autoregressive Distributive Lag model; CS-ARDL: Cross-sectional Autoregressive Distributive Lag model; AMG: Augmented mean group; FEVD: PMG-ARDL: Pooled Mean Group- Autoregressive Distributive Lag model; DOLS: Dynamic Ordinary Least Squares; FMOLS: Fully Modified Ordinary Least Squares; CCR: Canonical Cointegrating Regression; QQ: Quantile on quantile approach; CCEMG: Common correlated effects mean group; NARDL: Non-linear Autoregressive Distributive Lag model; POLS: Panel Ordinary Least Squares; GMM: Generalised Method of Moments; GLO: globalization; OECD: Organization for Economic Cooperation and Development; CCO_2_: consumption-based carbon emissions.

According to the assessment of related literature, while the effects of GDP, NREN, REN, and GLO on CO_2_ emissions in the MINT economies have been investigated, little or no attention has been giving to assessing these relationships on CCO_2_ emissions, particularly from the perspective of the MINT economies. Furthermore, although a substantial body of research has examined the impacts of the utilization of non-renewable energy on CCO_2_ emissions, studies on the joint effect of globalization and non-renewable energy, as well as the effect of globalization and renewable energy on consumption-based carbon emissions are limited. More significantly, no previous study has aimed to assess the effects of the joint impact of globalization and energy utilization on consumption-based carbon emissions in the MINT economies. Therefore, the outcomes provided in the literature above are not free from bias to some degree. Therefore, the present research aims to address the abovementioned gap in the prior literature through investigating the influence of renewable energy use and globalization on CCO_2_ as well as the role of economic growth in the MINT economies using a dataset from 1990 to 2018. This study will provide answers to the following questions:

• Is the EKC hypothesis valid for the MINT economies?
• Do non-renewable energy consumption and globalization have a combined influence on CCO_2_ emissions in the MINT economies?
• Do renewable energy consumption and globalization have a combined influence on CCO_2_ emissions in the MINT economies?
• What are the roles of globalization and renewable energy in reducing CCO_2_ emissions in the MINT economies?

## 3 Data, model specification, and estimation procedures

### 3.1 Data

This study compiled a balance panel dataset covering the time span of 1990–2018 for the MINT economies. Since data on consumption-based carbon emissions and globalization are not readily available, the research period begins in 1990 and runs through 2018. Consumption-based carbon emissions (environmental degradation) serves as the dependent variable, while its regressors are non-renewable energy use, globalization, economic growth, and renewable energy use. The series are transformed into their natural logarithms to ensure that they conform to normal distribution. Table 3 highlights the measurement and sources of the series used.

**Table 3:** Data description.

Variable	Symbol	Source
Consumption-based carbon emissions	CCO_2_	GCA
Economic growth	GDP	WDI
Non-Renewable energy Consumption	NREN	BP
Renewable energy Consumption	REN	WDI
Globalization	GLO	KOF

Note: GCA: Global Carbon Atlas; WDI- world development indicators; BP- British petroleum statistical review of world energy; KOF–KOF Swiss economic institute.

### 3.2 Model specification

According to the research of Adebayo & Rjoub [39] and Kirikkaleli & Adebayo [44], this study's econometric model is presented in Eq. (2):

(2) $$CCO_{2,\text{i}t}=\vartheta_{0}+\vartheta_{1}GDP_{it}+\vartheta_{2}NREN_{it}+\vartheta_{3}REN_{it}+\vartheta_{4}GLO_{it}+\varepsilon_{it}$$

Where: *t* specifies the considered period for this study (1990–2018); the cross-section (MINT economies) is depicted by *I*; ϑ denotes the coefficient's series; the error term is symbolized as *ε*. We expect that a positive relationship will exist between GDP and CCO_2 ._i.e., $\left(\vartheta_{1}=\frac{\partial CCO_{2}}{\partial GDP}>0\right)$. It has been shown in the prior literature that non-renewable and renewable energy use increase and decrease CCO_2_ separately. The signs for $\vartheta_{2}$ and $\vartheta_{3}$ are expected to be positive and negative i.e., $\left(\vartheta_{2}=\frac{\partial CCO_{2}}{\partial NREN}>0\text{ and }\vartheta_{3}=\frac{\partial CCO_{2}}{\partial REN}<0\right)$. Finally, for the relationship between globalization and CCO_2_ emissions, we anticipate a negative association. This suggests that $\vartheta_{4}$ is negative i.e $.,\left(\vartheta_{4}=\frac{\partial CCO_{2}}{\partial GLO}<0\right)$.

For the joint effect of energy utilization and globalization on CCO_2_ emissions in MINT economies, the interaction between globalization and both non-renewable and renewable energy utilization was examined, and their interaction terms were incorporated into the study's model, as shown in Eq. (3):

(3) $$CCO_{2,\text{i}t}=\vartheta_{0}+\vartheta_{1}GDP_{it}+\vartheta_{2}NREN_{it}+\vartheta_{3}REN_{it}+\vartheta_{4}GLO_{it}+\vartheta_{5}{\left(NREN*GLO\right)}_{it}+\vartheta_{6}{\left(REN*GLO\right)}_{it}+\varepsilon_{it}$$

We expect both $\vartheta_{5}$ and $\vartheta_{6}$ to be negative. Also, the EKC hypothesis, which is the square of GDP, was incorporated into the model, which is clearly presented in Eq. (4) as:

(4) $$CCO_{2,\text{i}t}=\vartheta_{0}+\vartheta_{1}GDP_{it}+\vartheta_{2}GDP_{it}^{2}+\vartheta_{3}NREN_{it}+\vartheta_{4}REN_{it}+\vartheta_{5}GLO_{it}+\vartheta_{6}{\left(NREN*GLO\right)}_{it}+\vartheta_{7}{\left(REN*GLO\right)}_{it}+\varepsilon_{it}$$

### 3.3 Estimation procedures

#### 3.3.1 Cross-sectional dependence (CSD) and slope heterogeneity tests

Cross-sectional dependence in panel data analysis is more likely to arise in this period of rising globalization and fewer trade barriers. Inability to resolve the challenge of cross-sectional dependency while asserting independence between cross-sections can result in inaccurate, biased, and erroneous assessments. This present research utilizes the [68] test for cross-sectional dependence. Equally, without verifying for a heterogeneous slope coefficient, the assumption of the coefficient of homogeneous slope will indeed generate erroneous estimation. Premised upon it, the [69] technique was used to assess the cross-section's slope heterogeneity; nevertheless, this method is a refined form of the [70] approach. Moreover, it is critical to analyze the slope homogeneity. The slope homogeneity testing equation is illustrated in Eq. (5) and Eq. (6):

(5) $${\tilde{\Delta }}_{SH}={\left(N\right)}^{\frac{1}{2}}{\left(2k\right)}^{-\frac{1}{2}}\left(\frac{1}{N}\tilde{S}-k\right)$$

(6) $${\tilde{\Delta }}_{ASH}={\left(N\right)}^{\frac{1}{2}}{\left(\frac{2k(T-k-1}{T+1}\right)}^{-\frac{1}{2}}\left(\frac{1}{N}\tilde{S}-2k\right)\;$$

Where: adjusted delta tilde is ${\tilde{\Delta }}_{ASH}$ and delta tilde is ${\tilde{\Delta }}_{SH}$.

#### 3.3.2 Panel unit root tests

The study deployed the [31] cross-sectional augmented IPS and ADF tests, also called the CADF and CIPS tests to detect the stationary features of the concerned variable. CADF is computed using the following Eq. (7):

(7) $$\Delta Y_{i,t}=\gamma_{i}+\gamma_{i}Y_{i,t-1}+\gamma_{i}{\bar{X}}_{t-1}+\overset{p}{\underset{l=0}{\sum }}\gamma_{il}\Delta \bar{Y_{t-l}}+\overset{p}{\underset{l=1}{\sum }}\gamma_{il}\Delta Y_{i,t-l}+\varepsilon_{it}$$

The lagged averages is depicted as ${\bar{Y}}_{t-1}$, and the averages first difference is depicted as $\Delta \bar{Y_{t-l}}$. For the CIPS computation, the average of the CADF needs to be derived, which is defined in Eq. (8) as follows:

(8) $$\hat{\text{CIPS}}=\frac{1}{\text{N}}\overset{\text{n}}{\underset{\text{i}=1}{\sum }}{\text{CADF}}_{\text{i}}$$

Where: CIPS: cross-sectional augmented IPS; CADF: cross-sectional augmented ADF. Hence, these unit root approaches are classified as second-generation unit root testing. In contrast to first generation unit root testing, these techniques generate reliable estimations in the condition of CSD.

#### 3.3.3 Panel cointegration test

Conventional panel cointegration approaches generate erroneous estimates whenever the parameters contain breaks and CSD. Westerlund [71] cointegration technique is used to assess the connection between CCO_2_ and the regressors in the long-run. The Westerlund [71] cointegration approach is computed in Eq. (9) as follows:

(9) $$\alpha i\left(L\right)\Delta y_{it}=y2_{it}+\beta_{i}\left(y_{it}-1-{\text{ά}}_{i}{\text{x}}_{\text{it}}\right)+{\text{λ}}_{\text{i}}\left(\text{L}\right){\text{v}}_{\text{it}}+{\text{η}}_{\text{i}}$$

Where. $\delta_{1i}=\beta_{i}\left(1\right){\hat{\vartheta }}_{21}-\beta_{i}\lambda_{1i}+\;\beta_{i}\hat{\vartheta }2_{i}\text{ ​and ​}y2_{i}=\;-\beta_{i}\lambda 2_{i}$

For the Westerlund cointegration approach, its test statistics is define in Eqs. (10), (11), (12), and (13), which is presented as follows:

(10) $$G_{t}=\;\frac{1}{N}\overset{N}{\underset{i-1}{\sum }}\frac{{\text{ά}}_{i}}{SE\left({\text{ά}}_{i}\right)}$$

(11) $$G_{\alpha }=\;\frac{1}{N}\overset{N}{\underset{i-1}{\sum }}\frac{\text{T}{\text{ά}}_{i}}{{\text{ά}}_{i}\left(1\right)}$$

(12) $$P_{T}=\;\frac{\text{ά}}{SE\left(\text{ά}\right)}$$

(13) $$P_{\alpha }=\;\text{Tά}$$

Where: the group T-statistics are ${\text{G}}_{\text{a}}$ and ${\text{G}}_{\text{t}}$ and the T-statistics for panel are ${\text{P}}_{\text{a}}$ and ${\text{P}}_{\text{t}}$. The hypothesis for this approach is the null hypothesis of no cointegration against the alternative hypothesis of cointegration.

#### 3.3.4 Common Correlated Effect Mean Group (CCEMG) and the augmented mean group (AMG) test

In the next step, the extent to which the determinants of consumption-based carbon emissions interact in the long-run was established by applying the Common Correlated Effect Mean Group (CCEMG) approach and Augmented Mean Group (AMG), which was developed by [30] approach. Prior research has relied on first-generation cointegration methods to examine the extent of the association. This first-generation cointegration approaches (such as ARDL, FMOLS, and DOLS, etc.) are based on the notion that the cross-sections are independent. This situation could cause their estimates to be unreliable. However, these approaches help in solving the issues of endogeneity, unobserved common factors, heterogeneous slope coefficients, non-stationarity, and cross-sectional dependence. The computation of the CCEMG in Eq. (14) is as follows:

(14) $$CC{O_{2}}^{i,t}=\theta^{1}{\bar{GDP}}^{i,t}+\theta^{1}{\bar{GDP}}^{2i,t}+\;\theta^{3}NREN^{i,t}+\theta^{4}{\bar{REN}}^{i,t}+\theta^{5}{\bar{GLO}}^{i,t}+\gamma^{i}+W^{i,t}+\varepsilon^{i,t}$$

## 4 Results and discussion

A summary of the study series is presented in Table 4. The outcomes of Table 4 show that NREN (3.872703) has the highest mean, which falls between 3.103554 and 4.338912, followed by GDP (3.653097), which falls between 3.127628 and 4.181561, then CCO_2_ (2.359355), which falls between 1.528312 and 2.771927, GLO (1.760292), which falls between 1.598991 and 1.858201, and REC (1.457985), which falls between 0.952542 and 1.948569. Moreover, the standard deviation results revealed that GLO is more consistent, followed by GDP, CCO_2_, REC, and EC. Furthermore, the skewness outcomes revealed that CCO_2_, NREN, GDP and GLO are negatively skewed, while the kurtosis outcomes showed that all the variables align with normal distribution.

**Table 4:** Descriptive statistics.

	CCO_2_	NREN	GDP	GLO	REN
Mean	2.359355	3.872703	3.653097	1.760292	1.457985
Median	2.474968	3.973000	3.729278	1.771369	1.478070
Maximum	2.771927	4.338912	4.181561	1.858201	1.948569
Minimum	1.528312	3.103554	3.127628	1.598991	0.952542
Std. Dev.	0.347994	0.373237	0.336593	0.062335	0.361741
Skewness	-0.955578	-0.481756	-0.132428	-0.455711	0.078067
Kurtosis	2.820078	1.701997	1.419665	2.508301	1.464796
Jarque-Bera	17.81030	12.63031	12.41010	5.183539	11.50927
Probability	0.000136	0.001809	0.002019	0.074887	0.003168

Moreover, we test for cross-section dependence (CSD) in the study variables by using the Breusch-Pagan LM, Pesaran scaled LM, Bias-corrected scaled LM and Pesaran CD tests. The outcomes of the CSD are depicted in Table 5. The research outcomes indicate that all the series have an issue of CSD. The significance of the CSD stems from the fact that economies are interrelated in the globally interconnected environment. This implies that any disruption in one country's underlying variables may spread to other nations. The variables are cross-sectionally dependent as a consequence of the spillover effects. Furthermore, we applied the slope homogeneity test developed by Pesaran & Yagamata [69], the outcomes of which are depicted in Table 6. They show that the MINT economies differ in terms of technical progress and growth. As a result, the results point to the possibility of heterogeneity in slope coefficients.

**Table 5:** CSD tests.

Tests	GDP	REN	NREN	GLO	CCO_2_
Breusch-Pagan LM	142.28∗	77.545∗	71.971∗	151.47∗	136.08∗
Pesaran scaled LM	39.341∗	20.653∗	19.044∗	41.994∗	37.552∗
Bias-corrected scaled LM	39.269∗	20.581∗	18.972∗	41.923∗	37.481∗
Pesaran CD	11.908∗	6.1636∗	5.7646∗	12.303∗	11.653∗

Note: ∗p < 0.01.

**Table 6:** Slope homogeneity outcomes.

Test	Model-1		Model-2		Model-3		Model-4	
	Value	P value	Value	P value	Value	P value	Value	P value
$\hat{\Delta }$	6.712∗	0.000	7.865∗	0.000	8.945∗	0.000	7.460∗	0.000
${\hat{\Delta }}_{\text{adjusted}}$	7.045∗	0.000	8.163∗	0.000	9.634∗	0.000	8.034∗	0.000

Note: ∗p < 0.01.

The current research takes a step further by assessing the variables' stationarity properties. In doing so, we applied the CIPS test initiated by Peseran et al. [72] to catch the series stationarity characteristics. The outcomes of the CIPS are depicted in Table 7. At level, REN and CCO_2_ are found stationary; however, after the first difference was taken REN, GDP, CCO_2_, GLO, and NREN are found stationary. This implies that the series have a mixed order of integration.

**Table 7:** CIPS.

Variables	Level	First Difference
CCO_2_	-2.777∗	-6.165∗
GDP	-1.907	-3.948∗
REN	-3.353∗	-6.068∗
NREN	-1.332	-5.684∗
GLO	-2.424∗∗	-5.259∗

Note: ∗ and ∗∗ represents p < 0.01 and p < 0.05 respectively.

We proceed by assessing the association between CCO_2_ emissions and the regressors in the long run. We applied the Westerlund cointegration initiated by [71] to capture the long-run interconnection between CCO_2_ emissions and the regressors. The outcomes from Table 8 show that in the four models, there is proof of a long-run association between CCO_2_ emissions and the independent variables. This infers that the null hypothesis of "no cointegration" is rejected. Therefore, in the four models, there is cointegration between CCO_2_ emissions and the regressors.

**Table 8:** Cointegration test Outcomes.

	Model-1	Model-2	Model-3	Model-4
Gt	-2.503∗∗∗	-2.967 ∗	-3.066∗	-2.502∗∗
Ga	-4.596	-5.621	-5.755	−4.575
Pt	-9.447∗∗	-10.421∗	-9.947∗	−9.333∗
Pa	-7.830∗∗∗	-9.657∗	-8.854 ∗∗	−-7.671∗∗∗

Note: ∗p < 0.01, ∗∗p < 0.05 and ∗∗∗p < 0.10.

After we confirmed the cointegration amongst the study series, we move forward by investigating the long-run interrelationship between CCO_2_ emissions and the regressors. In doing so, both the CCEMG and AMG long-run estimators are used. The outcomes of the CCEMG and AMG are both presented in Table 9.

**Table 9:** CCEMG and AMG outcomes.

Regressors	CCEMG				AMG			
	Model-1	Model-2	Model-3	Model-4	Model-1	Model-2	Model-3	Model-4
	Coefficient	Coefficient	Coefficient	Coefficient	Coefficient	Coefficient	Coefficient	Coefficient
GDP	0.8552∗	1.4750∗	0.9961∗	1.2066∗∗	1.1298∗	1.8277∗	0.7315∗	0.9398∗∗∗
NREN	0.6405∗	0.5641∗∗	1.9683∗∗∗	0.2539∗	0.2498∗	0.2001∗∗	-1.1439∗∗	0.1316∗
REN	-2.161∗∗	−0.880∗	−0.3854∗∗	-0.6255∗∗	-0.2407∗∗	-0.2407∗∗	-0.9325∗	-2.4568∗∗
GLO	-0.4498∗∗∗	−1.856∗∗∗	−1.5549∗	-1.1886	-0.4327∗	-0.4327∗	-2.2287∗∗	-1.0673
GDPSQ	-	-0.449∗∗∗	-	-	-	-0.6848∗	-	-
GLO∗NREN	-	-	-0.1833	-	-	-	-0.2219	-
GLO∗REN	-	-	-	-0.211∗∗	-	-	-	-1.453∗∗∗
Constant	1.0514	0.1113	1.8332	1.7706	0.9688	0.9005	0.2301	0.8175

Note: ∗p < 0.01, ∗∗p < 0.05 and ∗∗∗p < 0.10.

In the four models, the influence of GDP on CCO_2_ is positive and significant, which suggests that an upsurge in GDP in the MINT economies is accompanied by an upsurge in CCO_2_ emissions. The results suggest that the scale effect exceeded the composition and technique effects in these countries, indicating that economic growth is fostering environmental deterioration through the use of more energy and subsequent creation of more pollutants. This demonstrates that these economies place a higher priority on economic expansion than on damaging the environment. As a consequence, the environmental sustainability of these countries has deteriorated in the process of achieving more growth in the economy. Likewise, gross domestic product (GDP) is a metric of an economy's health and includes many elements such as investment, consumption, net exports, and government spending. Consumption accounts for the majority of GDP, and rising consumption is related to an upsurge in the emissions of CO_2_ [19]. Furthermore, when there is an upsurge in the income of the MINT countries, it is possible that not just the government, but also households and firms will consume more, which will trigger emissions of CO_2_. This research outcome is consistent with the study of He et al. [19] for Mexico between 1990 and 2018, which established a positive CCO_2_-GDP interrelationship. The outcome also validates the studies of [62] for Chile [39], for the MINT nations, and [26] for the BRICS nations.

Moreover, in Model-2, we found an adverse association between the squares of GDP and CCO_2_ emissions, which validates the inverted U-shaped interrelationship between growth and environmental degradation. This result shows that after a certain level of income is reached; environmental issues may be addressed by eco-efficiency laws, technological development, sustainable production, and consumption habits. It also demonstrates that the present policies of these nations are on the right course, as their economies are moving away from industries that are polluting towards industries using green technologies that emit less CO_2_ emissions. The negative and statistically significant CCO_2_-GDPSQ interconnection confirms the inverted U-shaped growth-emissions interconnection. As a result, economic development initially harms the environment before eventually benefitting it. This outcome complies with the works of [22] for WEMA nations and [73] for Chinese provinces, which validates the EKC hypothesis.

Furthermore, there is a positive NREN-CCO_2_ interrelationship, which is established by both CCEMG and AMG in the four models. This implies that an upsurge in NREN increase CCO_2_ emissions. The consumption of coal and crude oil has risen as a result of increasing energy use, resulting in higher CO_2_ emissions. Due to rapid growth in industry, manufacturing, and transportation in the post-liberalization era, the MINT nation's utilization of energy has increased substantially, and this energy-driven economic expansion has gradually impacted the quality of the environment adversely. This outcome complies with the studies of [74] for Thailand, and [2] for Argentina, who established that an upsurge in the consumption of nonrenewable energy triggers the emissions of CO_2_.

On the other hand, in the four models, renewable energy utilization and CCO_2_ emissions connection are negative, which demonstrates that REN can be utilized to abate CCO_2_ in the MINT nations. Since non-renewable energy usage is being replaced by the use of renewable alternatives (which is associated with the reduction of fossil fuel reliance), it can be argued that the utilization of renewables can aid in abating environmental degradation in the MINT nations. This suggests that lowering fossil fuel reliance through the use of renewable energy may be viewed as a viable way of mitigating CCO_2_ emissions in the MINT economies. The research of [27] for highly decentralized economies between 1990 and 2018 supports this finding. Furthermore, the studies of [75] for advanced economies [47], for Brazil [76], for China, and [2] for Argentina established that renewable energy use enhance quality of the environment.

Moreover, in the four Models, the effect of globalization (GLO) on CCO_2_ is found to be negative and significant, which infers that keeping other factors constant, a 1% upsurge in globalization causes a decrease in CCO_2_ emissions, as revealed by both CCEMG and AMG in Table 9. This demonstrates that globalization helps in curbing CCO_2_ emissions in the MINT nations. With the globalization index as well as CO_2_ levels both increasing in recent years, this study challenges the notion that globalization produces higher CO_2_ emissions levels. This study outcome corroborates the findings of [2] in Argentina for the period between 1980 and 2018, who established a negative emissions-globalization interconnection. Moreover, this outcome also complies with the study of [19] for Mexico between 1990 and 2018, which established that an upsurge in globalization mitigates CCO_2_ emissions. However, this outcome contradicts the studies of [20] for South Africa and [24] for Turkey, who established a positive emissions-globalization interrelationship.

To further understand the rationale for such a perplexing result, we examined the joint effects of globalization and nonrenewable energy consumption on CCO_2_ emissions, and the outcomes revealed a negative and insignificant impact, suggesting that the combined influence of globalization and nonrenewable energy consumption does not abate CCO_2_ emissions in the MINT nations, as shown in Model-3. Furthermore, in Model-4, we assess the joint effect of globalization and renewable energy consumption on CCO_2_ emissions. The results indicate that both renewable energy and globalization jointly curb CCO_2_ emissions.

The current research proceeds by investigating the causal interconnection between CCO_2_ and the regressors for the MINT nations between 1990 and 2018. The outcomes of the causality test are depicted in Table 10. The results show evidence of a bidirectional causal interrelation between NREN and CCO_2,_ which implies that both NREN and CCO_2_ can predict each other in the MINT nations. This outcome complies with the study of He et al. [19] for Mexico, which established a two-way causal association between NREN and CCO_2_. Moreover, a unidirectional causal impact was established from GDP to CCO_2,_ which is in accordance with the studies of Khan et al. [41] for nine oil-exporting nations and [39] for the MINT nations. In addition, there is evidence of a causality from REN to CCO_2_ which aligns with the studies of [26] for the BRICS nations and Khan et al. [41] for nine oil-exporting nations. Lastly, we observed a one-way causal interconnection from globalization to CCO_2,_ which corroborates the studies of [19] for Mexico [56], for nine oil-exporting nations, and [26] for the BRICS nations.

**Table 10:** Dumitrescu hurlin panel causality outcomes.

Causality Direction	W-Stat.	Zbar-Stat.	Prob.	Decision
NREN → CCO_2_	4.6049	1.9776	0.0480∗∗	Bidirectional Causality
CCO_2_ → NREN	6.3336	3.3989	0.0007∗	
GDP → CCO_2_	10.838	3.1658	0.0015∗	Unidirectional Causality
CCO_2_ → GDP	4.9449	0.1887	0.8503	
GLO → CCO_2_	2.1915	-0.0069	0.9944	Unidirectional Causality
CCO_2_ → GLO	4.3310	1.75233	0.0797∗∗∗	
REN → CCO_2_	4.6826	1.8651	0.0705∗∗∗	Unidirectional Causality
CCO_2_ → REN	3.9016	1.3992	0.1617	

Note: ∗p < 0.01, ∗∗p < 0.05 and ∗∗∗p < 0.10.

## 5 Conclusion and policy implications

Environmental contamination has become a prominent topic of debate all around the world. As a result, countries across the globe are attempting to establish and implement laws that will allow them to achieve economic growth without damaging the environment. Reducing emissions is critical for developing nations such as the MINTs (Mexico, Indonesia, Nigeria, and Turkey), as these countries are projected to contribute significantly to world output and, as a result, are anticipated to contribute to the high percentage of global GHG emissions. Against this backdrop, the current research assesses the influence of renewable energy consumption and globalization on consumption-based carbon emissions (CCO_2_) as well as the role of globalization and nonrenewable energy consumption in the MINT nations utilizing panel data covering the period from 1990 to 2018. The study utilized the CSD, CCEMG, Westerlund cointegration, AMG, and panel causality approaches to assess these interconnections. The outcomes from the cointegration test revealed a long-run association among the variables. Furthermore, the outcomes from both CCEMG and AMG revealed that economic growth and energy utilization trigger CCO_2_ emissions in MINT nations. However, globalization and renewable energy utilization helps to mitigate environmental degradation in the MINT nations. Moreover, the joint effect of globalization and renewable energy consumption helps to abate environmental degradation, while the joint effect of globalization and nonrenewable energy consumption does not play a vital role in curbing environmental degradation. Lastly, the causality outcomes disclosed that globalization, economic growth, non-renewable energy utilization and renewable energy utilization can predict CCO_2_ emissions. Thus, policy channeled towards all the regressors will have a significant effect on CCO_2_ emissions.

In the context of these outcomes, numerous policy-level recommendations can be suggested for the MINT economies to simultaneously achieve economic and environmental wellbeing. First, the study suggests that, in order to mitigate the impact of economic development on CCO_2_ emissions, domestic consumption levels should be addressed, particularly in those sectors that are more energy-intensive and cause CO_2_ emissions to rise. Secondly, MINT countries must minimize their dependence on non-renewable energy to fulfill domestic energy needs. Since renewable energy consumption is important for reducing environmental degradation, supportive policies must be established and put in place to eliminate the conventional hurdles that have stymied the adoption of renewable energy in the MINT countries.

Thirdly, although globalization helps in abating the degradation of the environment in the MINT nations, it is critical to guarantee that the globalization-induced increase in energy demand is met with renewable energy. In this sense, the MINT countries might seek to exchange renewable energy with their neighbors, thereby enhancing the good ecological results connected with trade globalization. At the same time, the governments of the MINT countries should consider attracting FDI to help expand their renewable energy sectors. Financial globalization-induced inflow of FDI is likely to result in technology spillover, easing the technological restrictions that have hampered renewable energy implementation in the MINT countries. Finally, the MINT countries must accelerate their economic growth rates, particularly via the use of cleaner and renewable energy supplies, in order to attain the economic expansion benchmark beyond which both environmental and economic progress can be achieved concurrently. As a result, it is once again suggested that the MINT nations minimize their reliance on fossil fuels and adapt their manufacturing processes to make them more ecologically friendly. It is believed that the implementation of these measures will aid the MINT nations in meeting their environmental sustainability obligations.

The study's time frame was shortened due to a lack of relevant data. Furthermore, due to data constraints, we were unable to include additional important macroeconomic factors in our models. This research can be expanded in the future to examine the effects of other aspects of globalization on the MINT nations' CCO_2_ emissions as well as other environmental sustainability metrics.

## Declarations

### Author contribution statement

Tomiwa Sunday Adebayo, Abraham Ayobamiji Awosusi, Husam Rjoub: Conceived and designed the experiments; Analyzed and interpreted the data; Contributed reagents, materials, analysis tools or data; Wrote the paper.

Ephraim Bonah Agyekum, Dervis Kirikkaleli: Analyzed and interpreted the data; Contributed reagents, materials, analysis tools or data; Wrote the paper.

### Funding statement

This research did not receive any specific grant from funding agencies in the public, commercial, or not-for-profit sectors.

### Data availability statement

Data included in article/supplementary material/referenced in article.

### Declaration of interests statement

The authors declare no conflict of interest.

### Additional information

No additional information is available for this paper.

## Footnotes

1 https://ukcop26.org/.

2 Consumption-based carbon emissions capture lifecycle and direct GHG emissions from services and goods and assigns GHG emissions to the end consumer of those services and goods rather than the original producers the emissions, while Territory-based emissions, also known as production emissions, are those that occur inside a nation's borders and include exports but exclude imports.
